# An Approach for Characterizing and Comparing Hyperspectral Microscopy Systems

**DOI:** 10.3390/s130709267

**Published:** 2013-07-19

**Authors:** Naga S. Annamdevula, Brenner Sweat, Peter Favreau, Ashley S. Lindsey, Diego F. Alvarez, Thomas C. Rich, Silas J. Leavesley

**Affiliations:** 1 Department of Chemical and Biomolecular Engineering, University of South Alabama, 150 Jaguar Dr., SH 4129, Mobile, AL 36688, USA; E-Mails: nsa801@jagmail.southalabama.edu (N.S.A.); ws1001@jagmail.southalabama.edu (B.S.); pff601@jagmail.southalabama.edu (P.F.); 2 Department of Pharmacology, University of South Alabama, 5851 USA Dr. N., Mobile, AL 36688, USA; E-Mails: aes901@jagmail.southalabama.edu (A.S.L.); dalvarez@southalabama.edu (D.F.A.); trich@southalabama.edu (T.C.R.); 3 Center for Lung Biology, University of South Alabama, 5851 USA Dr. N., Mobile, AL 36688, USA; 4 Department of Internal Medicine, University of South Alabama, 5851 USA Dr. N., Mobile, AL 36688, USA; 5 College of Engineering, University of South Alabama, 150 Jaguar Dr., Mobile, AL 36688, USA

**Keywords:** hyperspectral imaging, spectral analysis, microscopy, confocal, widefield, signal to noise ratio, photobleaching

## Abstract

Hyperspectral imaging and analysis approaches offer accurate detection and quantification of fluorescently-labeled proteins and cells in highly autofluorescent tissues. However, selecting optimum acquisition settings for hyperspectral imaging is often a daunting task. In this study, we compared two hyperspectral systems—a widefield system with acoustic optical tunable filter (AOTF) and charge coupled device (CCD) camera, and a confocal system with diffraction gratings and photomultiplier tube (PMT) array. We measured the effects of system parameters on hyperspectral image quality and linear unmixing results. Parameters that were assessed for the confocal system included pinhole diameter, laser power, PMT gain and for the widefield system included arc lamp intensity, and camera gain. The signal-to-noise ratio (SNR) and the root-mean-square error (RMS error) were measured to assess system performance. Photobleaching dynamics were studied. Finally, theoretical sensitivity studies were performed to estimate the incremental response (sensitivity) and false-positive detection rates (specificity). Results indicate that hyperspectral imaging assays are highly dependent on system parameters and experimental conditions. For detection of green fluorescent protein (GFP)-expressing cells in fixed lung tissues, a confocal pinhole of five airy disk units, high excitation intensity and low detector gain were optimal. The theoretical sensitivity studies revealed that widefield hyperspectral microscopy was able to detect GFP with fewer false positive occurrences than confocal microscopy, even though confocal microscopy offered improved signal and noise characteristics. These studies provide a framework for optimization that can be applied to a variety of hyperspectral imaging systems.

## 1. Introduction

Hyperspectral imaging was first introduced in the remote sensing field for satellite imagery analysis [1]. More recently, hyperspectral imaging has gained importance in biological imaging applications, where it has found utility for separation of fluorescence emission signals from multiple fluorophores [2,3], separation of fluorescent label and autofluorescence emission signals [4], *in-vivo* imaging [5], and FRET analysis [6–8]. In our previous studies, we showed that hyperspectral imaging microscopy and analysis offers accurate detection and quantification of fluorescently-labeled cells in highly autofluorescent tissues [9–11]. Hyperspectral imaging can be performed on various system configurations: widefield fluorescence microscopy [9,12], confocal microscopy [13-15], and *in-vivo* fluorescence imaging [5,16,17]. Each system configuration has its own advantages and disadvantages in performing hyperspectral imaging assays. Of particular importance is the scanning method (sequential, push-broom, or raster-scanning), the method for separating or isolating specific wavelength bands (filter wheels, tunable filters, dispersive elements, interferometry), and the detector sensitivity and noise characteristics. While some hyperspectral imaging technologies are still in the developmental stage, several hyperspectral imaging platforms are currently available for widefield and confocal microscopy. Unfortunately, there is little quantitative information available to aid researchers in selecting an appropriate system or adjusting system parameters for optimal performance. Hence, there is a need to understand how spectral filtering and detector characteristics affect the sensitivity and specificity of hyperspectral image acquisition and spectral image analysis approaches, and how to optimize the parameters of a hyperspectral microscopy system for different, specific experimental preparations. Though comparisons have been made between wide-field and confocal single band fluorescence microscopy [18], single band widefield and hyperspectral widefield microscopy [9], and different hyperspectral imaging systems [19], an approach for quantitative comparison between different hyperspectral imaging systems has not been demonstrated.

The most important considerations of hyperspectral imaging systems are the ability to detect specific fluorescence signals (sensitivity) and to discriminate among multiple fluorophores (specificity). In this work, we demonstrate that the detection sensitivity and specificity of hyperspectral imaging and analysis approaches depend on both the signal and noise characteristics of the detector and properties of the system as a whole. In addition, the sensitivity and specificity may vary over the course of an experiment, due to photobleaching and other kinetic interactions. The goal of this work is to develop a methodology for comparing hyperspectral microscopy systems and to demonstrate this methodology through a quantitative comparison of widefield and confocal hyperspectral fluorescence microscope systems. We used a previously-demonstrated hyperspectral assay for detecting green fluorescent protein (GFP)-expressing pulmonary microvascular cells (PMVECs) in lung tissue slices [9]. Lung tissue presents high autofluorescence emission with a peak emission wavelength near that of GFP, making it prohibitively difficult to detect GFP using non-spectral imaging techniques. Similar fields-of-view from the same sample were imaged on both the widefield and confocal hyperspectral microscope systems to compare the signal-to-noise characteristics, sensitivity, and specificity of each system for identifying GFP-expressing cells. To better compare both systems, we performed a parametric analysis in which individual system parameters were varied and the signal and noise characteristics and accuracy of linear unmixing were evaluated for each hyperspectral imaging system. System parameters included confocal pinhole diameter [20,21], type of detector and detector gain [21], type of illumination source and illumination intensity [21], and photobleaching time [22].

The results of this study indicate that selecting the correct values for each parameter is important for optimizing signal-to-noise characteristics and linear unmixing accuracy. In addition, the rate of photobleaching of different fluorophores can vary (differential photobleaching). Care should be taken when choosing system parameters in live-cell or live-tissue assays where a compromise must be made between the signal detection and photobleaching rate. For this assay, we found that the hyperspectral widefield microscope was more specific (produced fewer false positives) than the hyperspectral confocal microscope, even though the confocal microscope produced images with improved signal-to-noise characteristics at comparable system settings. Our study offers a framework for selecting appropriate values for hyperspectral imaging system parameters and for characterizing hyperspectral imaging systems. In addition, this approach can be extended to compare other hyperspectral imaging systems and to select optimal operating parameters for a particular assay.

## 2. Methods

### 2.1. Cell, Animal, and Tissue Preparations

Pulmonary microvascular endothelial cells (PMVECs) were isolated from CD rats as described previously [23]. Briefly, CD rats were anesthetized and subjected to a thoracotomy, and the lungs were excised. The tissue was dissected and placed in a 60 mm dish containing cold DMEM (4 °C). Tissue was digested with type II collagenase and PMVECs were characterized by flow cytometry after cell expansion (passages 3–7). After isolation and characterization, PMVECs were transduced with a lentivirus vector encoding green fluorescent protein (GFP) plasmid under a CMV promoter for 24 h. PMVECs with and without GFP were used as GFP-positive and GFP-negative cells, respectively, and were prepared as a monolayer on 25 mm round glass coverslips. Cover slips were mounted in a circular holder, stained with Hoechst 33342 (20 μg/mL concentration), bathed in extracellular buffer, and incubated at 37 °C for 20 min prior to imaging.

*P.aeruginosa* strain PA 103 was used to induce pneumonia and cause acute lung injury in rats. Rats were anesthetized and received an intra-tracheal injection of PA103 at the LD_50_ in 150 μL saline solution (37 °C) through a 27 gauge needle directly caudal to the thyroid gland. GFP-labeled PMVECs were intravenously infused into the rats at 10 × 10^3^ cells/100 g of body weight. After 1 week, the rats were subjected to thoracotomy and the lungs were excised. The portions of lungs were fixed at a constant volume, immersed in formalin, and embedded in paraffin. The tissues were cut into 5 μm thick sections and were placed on glass slides for imaging. Animal and care procedures conformed to the Institutional Animal Care and Use Committee (IACUC).

### 2.2. Hyperspectral Microscope Systems

**Figure 1.**
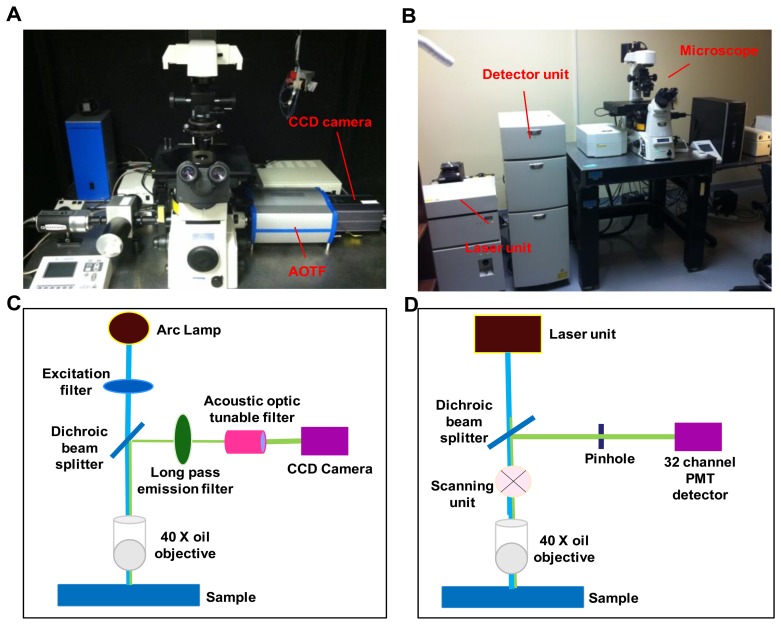
Photographs and light path of the hyperspectral imaging systems used in this study. (**A**) hyperspectral widefield fluorescence microscope; (**B**) hyperspectral confocal fluorescence microscope; (**C**) schematic describing the light path in the widefield system; (**D**) schematic describing the light path in the confocal system.

A comparative study was performed on two hyperspectral fluorescence microscopes: An inverted widefield microscope and an inverted confocal microscope. The instrumental setup for both the imaging systems is shown in Figure 1.

#### 2.2.1. Widefield Hyperspectral Fluorescence Microscope

The widefield hyperspectral imaging system used in this study consisted of an inverted fluorescence microscope (TE2000-U) equipped with a 40x oil immersion objective (S Fluor, 40×/1.30 Oil, DIC H/N2, Nikon Instruments, Melville, NY, USA), Xenon arc lamp (Lambda DG-4, Sutter Instruments, Novato, CA, USA), a variable bandwidth AOTF (HSi-300, Chromodynamics, Orlando, FL, USA), and an electron-multiplied charge coupled device (EMCCD) camera (Rolera EM-C^2^, QImaging, Inc., Surrey, BC, Canada) for image detection. The widefield microscope system provided a spatial sampling of 0.136 μm/pixel. A 360/40 nm excitation filter (D360/40, Chroma Technology Corp., Bellows falls, VT, USA) was used to excite Hoechst 33342 and a 430/24 excitation filter (ET 430/24, Chroma Technology Corp., Bellows falls, VT, USA) was used to excite autofluorescence and GFP. Note that 430 nm is lower than the peak excitation wavelength for GFP. However, this excitation wavelength was selected in order to allow the full emission spectrum of GFP to be scanned using hyperspectral imaging. A similar approach was also employed for confocal hyperspectral imaging, as described below. An emission wavelength range of 462 nm to 648 nm, with a 6 nm increment was used for all hyperspectral image sets. Micro-manager software was used to control the AOTF and EMCCD camera.

#### 2.2.2. Confocal Hyperspectral Fluorescence Microscope

The hyperspectral confocal imaging system was A1R spectral confocal microscope. The spectral detector consists of a 32-channel photomultiplier tube (PMT) array and three diffraction gratings (allowing 2.5 nm, 6 nm, or 10 nm spectral sampling increments). The same 40× objective that was used for the widefield system was also used on the confocal system, for consistency. The confocal microscope system provided a spatial sampling of 0.62 μm/pixel. Dual laser excitation at (405 nm and 458 nm) was used to simultaneously excite Hoechst, autofluorescence, and GFP. Unlike the widefield system, the wavelength spacing and bandwidth of the confocal microscope were fixed by the diffraction grating selected. Each spectral image consisted of 32 bands with a wavelength range of 462-648 nm, in 6 nm increments. NIS Elements software (Nikon Instruments, Inc., Melville, NY, USA) was used to acquire all confocal images.

### 2.3. Excitation Intensity Measurements

The excitation intensity, or optical irradiance, provided by each system at the image plane was measured using a high-sensitivity fiber-coupled spectrometer (QE65000, Ocean Optics, Inc., Dunedin FL, USA) connected to an integrating sphere (FOIS-1, Ocean Optics, Inc., Dunedin, FL, USA). The spectrometer was calibrated using a NIST-traceable light source (LS-1-CAL-INT, Ocean Optics, Inc., Dunedin, FL, USA), as per the manufacturer's standard operating procedure. The absolute irradiance spectrum was then measured by placing the integrating sphere on the stage, immediately above the objective. For the widefield system, the irradiance spectrum was sampled separately for each excitation filter. The irradiance spectrum was then integrated over the width of the excitation band ± 40 nm to each side of the center wavelength (to ensure that the cut-off of the band was included in measurements). For the confocal system, the irradiance spectrum was integrated separately, with a 10 nm band for each laser line.

### 2.4. System Calibration—Flat Spectral Correction

The widefield hyperspectral imaging system was calibrated using a NIST-traceable light source, as described previously [9]. Briefly, sample blanks were prepared (a blank slide and coverslip mounted with antifade fluorescence mounting medium for a tissue blank and a 25 mm round coverslip with extracellular buffer for cell-suspension blank). Background (dark) and control (bright) images were acquired using identical acquisition settings as those for the tissue and cell samples. The dark image was collected with the excitation shutter open. The bright image was acquired similarly to the dark image, but with the addition of the NIST-traceable light source placed on the stage adjacent to the sample. The spectral transfer function of the system was then calculated as:

(1) $$TF(\lambda )=\frac{I_{\text{Bright}}\;(\lambda )-I_{\text{Dark}}\;(\lambda )}{I_{\text{Lamp}}\;(\lambda )}$$

where *I_Bright_* is the spectrum measured from the bright image, *I_Dark_* is the spectrum measured from the dark image, and *I_Lamp_* is the NIST-traceable lamp spectrum (supplied by the manufacturer) at each wavelength, *λ*. A correction coefficient (CC (*λ*)) was calculated as the reciprocal of the spectral transfer function (1/TF (*λ*)). All spectral images acquired from the widefield microscope system were corrected by subtracting the dark spectrum and then multiplying the correction coefficient:

(2) $${\text{I}}_{\text{Corrected}}\;(\lambda )=({\text{I}}_{\text{Raw}}\;(\lambda )-{\text{I}}_{\text{Dark}}\;(\lambda ))\times \text{CC}(\lambda )$$

where *I_Raw_* is the raw spectrum for each pixel in the image, *CC* is the correction coefficient, and *I_Corrected_* is the corrected image. Spectral correction was not performed on the confocal system because the spectral detector is a NIST-traceable detector.

### 2.4. Spectral Image Analysis

Images acquired on both systems were converted to single band sequential image format using MATLAB software (MathWorks, Inc., Natick, MA, USA).

#### 2.4.1. Spectral Library

A spectral library was constructed using pure spectra for Hoechst and GFP and an average spectrum from autofluorescence. Spectral images from a monolayer of cultured GFP-expressing PMVECs were used for the pure GFP spectrum. Similarly, spectral images from a monolayer of control PMVECs (PMVECs not expressing GFP) stained with Hoechst were used for the pure Hoechst spectrum. Images of unlabeled lung tissue were used for the autofluorescence spectrum. In each case, after the background subtraction of spectral images, a region of interest (ROI) was defined using intensity thresholding of the total fluorescence emission (sum of all wavelength bands). Spectra for each end-member (GFP, Hoechst, and autofluorescence) were measured as the pixel-averaged spectrum for all pixels within the ROI.

#### 2.4.2. Linear Unmixing

We used ENVI software (ITT Visual Information Solutions, Boulder, CO, USA) for spectral analysis and linear spectral unmixing. The spectral library was used to linearly unmix the GFP, autofluorescence and Hoechst from the mixed images. The unmixed images were saved as tiff files and NIS elements software was used to handle and process the unmixed images. Excel (Microsoft Corporation, Redmond, WA, USA) was used to handle and manipulate the extracted spectral data.

### 2.5. Parametric Studies

We performed parametric studies on both the widefield and confocal systems to optimize the system parameters. For this study, confocal pinhole diameter, laser power, and PMT gain were selected on the confocal microscope. Arc lamp intensity and EMCCD camera gain were selected on the widefield microscope. These parameters were varied to characterize both hyperspectral imaging systems. A range of values was selected for each parameter. Spectral images were acquired at each setting of each selected parameter. The signal-to-noise ratio (SNR) and root-mean-square (RMS) error were calculated to optimize the selected system parameters. Experimental procedures for varying each parameter are described below.

#### 2.5.1. Confocal Pinhole Diameter

The confocal pinhole diameter was varied from 0.7 to 13.7 Airy disk units (13.1 to 255.4 μm ) in the following values: 0.7, 1, 3, 5, 7, 9, 11, and 13.7 Airy disk Units. At each pinhole setting, five sequential images were collected. The signal-to-noise ratio (SNR) was calculated for each wavelength band at each pixel, as described below (Section 0).The SNR was calculated for several cases: entire spectral image, selected regions-of-interest (ROIs) within spectra images, entire unmixed images, and selected ROIs within unmixed images. The RMS error associated with linear spectral unmixing was also calculated.

#### 2.5.2. Laser Intensity and Photomultiplier Tube (PMT) Gain

The effects of laser power and PMT gain on hyperspectral imaging analysis were studied for the spectral confocal system. As described above, each sample was excited simultaneously using 405 and 458 nm lasers. When varying total laser power, the ratio between the two lasers was kept constant. The PMT gain was adjusted with variation of laser power to maintain constant signal strength at 510 nm. Laser intensity and PMT gain values are summarized in Table 1. Similar to the confocal pinhole diameter study, five sequential images were acquired at each setting and the SNR and RMS error were calculated.

#### 2.5.3. Arc Lamp Intensity and Electron-Multiplied Charge Coupled Device (EMCCD) Camera Gain

We performed experiments analogous to adjusting the laser power and PMT gain on the spectral confocal system on the spectral widefield system by adjusting the arc lamp intensity and EMCCD camera gain. Samples on the widefield microscope were excited sequentially using 430 nm and 360 nm excitation filters. The arc lamp intensity was adjusted to 33, 50, and 100% of the maximal output. At each setting, using the 430 nm excitation filter, the EMCCD gain was adjusted accordingly to maintain constant signal intensity at 510 nm emission. As above, 5 sequential images were acquired at each setting and SNR and RMS error were calculated for each experimental setting.

#### 2.5.4. Signal-to-Noise Ratio (SNR)

The SNR was used to characterize instrument performance for each case described above. Hence, for a particular setting, we obtained 32 SNR values, which were then averaged to obtain a composite SNR of the spectral image. The SNR for unmixed images and for ROIs was also calculated. SNR calculations were performed using MATLAB. The SNR was calculated from the mean and standard deviation of sequential images, for each wavelength band, at each pixel:

(3) $$\text{SNR}=\frac{\mu_{\text{signal}}}{\sigma_{\text{signal}}}$$

where *μ_signal_* is the mean signal value for a given wavelength at a single pixel and *σ_signal_* is the standard deviation.

#### 2.5.5. Root-Mean-Square Error and Percent RMS Error

The root–mean–square (RMS) error, as measured between a spectral image and the sum of the corresponding unmixed images, was used as a measure of the error associated with spectral analysis. We used ENVI software for both linear unmixing and to measure RMS error. RMS error is given by:

(4) $$\text{RMSerror}=\sqrt{\overset{1}{\;}/\underset{n}{\;}{\sum \left(x_{\text{measured}}-x_{\text{fit}}\right)}^{2}}$$

where *n* is the number of wavelength bands, *x_measured_* is the intensity value of the measured pixel in the image and *x_fit_* is the intensity value of the pixel in the fitted curve. The RMS error was pixel-by-pixel measurement. Using RMS error, percent RMS error (RMS error/RMS signal) was calculated for all the spectral images and used to optimize the system parameters and to characterize the imaging systems.

#### 2.5.6. Photobleaching Analysis

Photobleaching studies were performed to assess the sensitivity of GFP, Hoechst, and autofluorescence to extended exposure times—often required for acquiring hyperspectral image data. A photobleaching study was performed on both the widefield and confocal systems. We selected a field-of-view (FOV) containing Hoechst, GFP, and autofluorescence was excited continuously for 1 h while acquiring images. On the widefield system, images were acquired every 5 minutes. Images were acquired every 30 s. on the confocal system. Only images at 5-minute intervals were analyzed to ensure consistency between systems. Spectral images at each time point were then unmixed as described above.

#### 2.5.7. Theoretical Sensitivity Studies

We assessed the theoretical sensitivity of both systems by adding varying amounts of known GFP signal to spectral images containing only autofluorescence signal (negative control tissues) as described previously [9]. As the hyperspectral confocal system features a 12-bit detector (multi-anode PMT) and the hyperspectral widefield system features a 16-bit detector (EMCCD camera), both images were linearly scaled to 16-bit space, to allow comparison of intensity levels. The spectrum of GFP was then added to a small region of the image post-acquisition, using a custom MATLAB script. The intensity of the GFP spectrum that was added was varied to measure the response of the linear unmixing algorithm to increasing signal strength for a single component, given that the autofluorescence signal is also present in the image. To accomplish this, an ROI was selected for analysis—in this case a 30 × 30 pixel square region. The GFP spectrum, at a specified peak intensity, was added to the ROI. Peak intensities of GFP signal added ranged from 0 to 1,000 in increments of 100. Spectral images with added GFP signal were linearly unmixed and the GFP abundance was plotted as a function of the GFP signal added to the image. This plot was used to determine the sensitivity response. A minimum detection threshold for GFP was defined using the sensitivity plot, which then was used to determine whether a pixel was GFP-positive or -negative. For these studies,the GFP detection threshold was defined as the 98.5% quantile (of abundance) for the image with 0% GFP added. Hence, the minimum GFP detection threshold is the 98.5% quantile value of the 30 × 30 pixel square region (900 pixels) with 0% GFP added. The number of GFP-positive pixels was then calculated using the minimum detection threshold for each amount of GFP signal added to the spectral image.

## 3. Results

### 3.1. Spectral Correction

**Figure 2.**
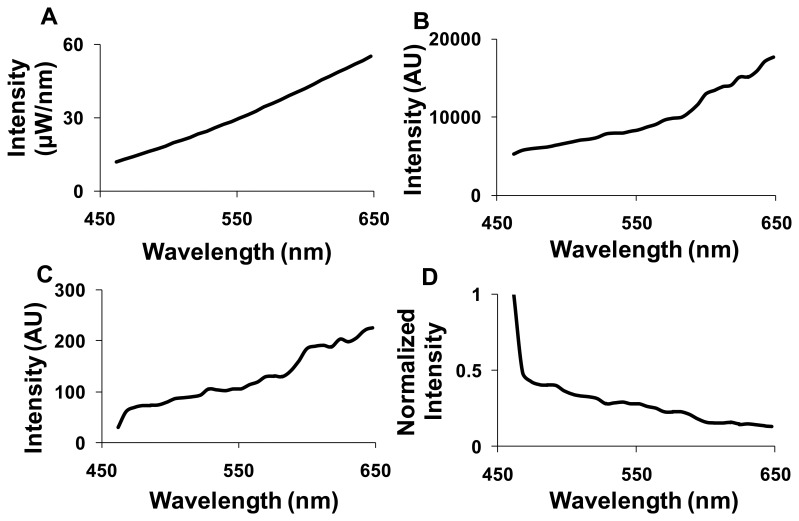
Flat spectral correction was used to compensate wavelength dependent-attenuation of fluorescence emission. (**A**) The spectrum of the US National Institute of Standards & Technology (NIST)-traceable lamp; (**B**) the spectrum of the NIST-traceable lamp as measured by the hyperspectral microscope; (**C**) the transfer function of the hyperspectral microscope; (**D**) the correction coefficient to perform flat-field correction.

Spectral correction was performed for the images acquired using the hyperspectral widefield fluorescence microscope to account for wavelength-dependent attenuation. A NIST traceable lamp was used to calibrate the widefield hyperspectral imaging system (Figure 2A). The output of the NIST-traceable lamp (Figure 2B) and the background spectrum were measured using the hyperspectral widefield microscope. These spectra were used to calculate the transfer function of the system (Figure 2C). The inverse of the transfer function was then used as a correction coefficient (Figure 2D). All further spectral images from the widefield system were background subtracted—using a blank slide or blank region of interest—and then corrected for wavelength-dependent attenuation using the correction coefficient. The hyperspectral confocal microscope uses a NIST-traceable detector; hence, we did not perform additional wavelength calibration. Background subtraction was still performed prior to image analysis.

### 3.2. Spectral Library

**Figure 3.**
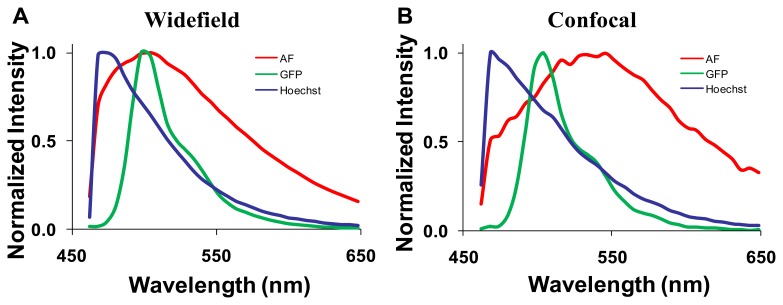
Spectral libraries used for linear spectral unmixing contained three components—lung autofluorescence (AF), green fluorescent protein (GFP), and Hoechst. (**A**) The spectral library acquired using the hyperspectral widefield microscope; (**B**) the spectral library acquired using the hyperspectral confocal microscope.

Spectra for GFP, Hoechst, and autofluorescence were obtained from cultured GFP-expressing PMVECs, Hoechst-labeled control wild-type PMVECs, and unlabeled lung tissue. These spectra were obtained from each microscope system and saved as spectral libraries (Figure 3). The spectra for GFP and Hoechst were similar on both microscope systems. However, there was a difference between the peak emission wavelength of autofluorescence obtained on each system (505 nm on the widefield and 545 nm on the confocal). The differences were attributed to the difference in excitation wavelengths used for each system (430/24 nm excitation band using the widefield system and 405 and 458 nm excitation using the confocal system).

### 3.3. Parametric Studies

**Figure 4.**
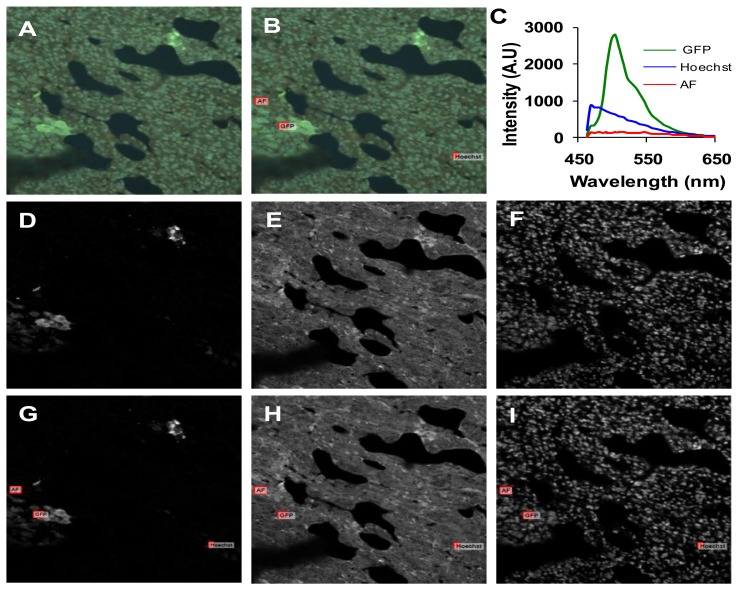
A representative image of a 10 μm lung cryoslice containing green fluorescent protein (GFP)-expressing cells and labeled with Hoechst. (**A**) False colored representation of spectral image data acquired on the confocal system. The signal-to-noise ratio (SNR) was calculated for the entire field of view (FOV); (**B**) Regions of interest (ROIs) corresponding to high emission of each fluorophore in the image were selected and the SNR was calculated for each ROI; (**C**) Mean fluorescence intensity corresponding to the ROI of each fluorophore averaged in five sequential images; (**D**–**F**) Linear unmixing of the image shown in panel A results in an abundance image for each endmember: GFP (D), autofluorescnece (E), and Hoechst (F). The SNR was calculated for each unmixed image using the entire FOV; (**G**–**I**) The same ROIs shown in panel B were applied to each unmixed image and the SNR was calculated for each ROI in each unmixed image.

We performed parametric sensitivity studies to characterize and compare the two imaging systems. The confocal pinhole diameter, laser power, and PMT gain were adjusted on the confocal microscope system, while the arc lamp intensity and EMCCD gain were adjusted on the widefield microscope system. The SNR and RMS error were calculated to evaluate the effects of each parameter on system response. The SNR was calculated to evaluate the effects of each parameter on system response. SNR for the whole image, for selected regions of interests (ROIs), and for unmixed images was calculated. Figure 4 shows how SNR was extracted for each of these cases. To mitigate the effects of spatially averaging weak fluorescence emission regions with strongly emitting regions, we used a single ROI localized around a high emission region of each fluorophore and applied this ROI to 5 sequential images, allowing calculation of the SNR in the ROI. In addition, we measured the mean fluorescence intensity in sequential images for an ROI of each fluorophore (Figure 4C). The coefficient of variation (CV) for each fluorophore was also calculated.

#### 3.3.1. Confocal Pinhole Diameter Analysis

**Figure 5.**
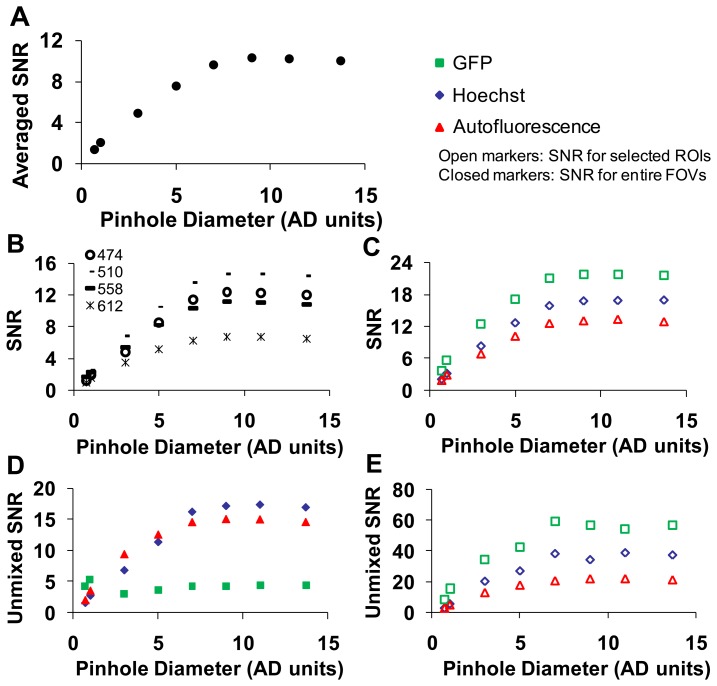
Signal-to-noise characteristics for the confocal system. (**A**) The signal-to-noise ratio(SNR) averaged over a field of view and over all wavelengths; (**B**) the SNR averaged over an entire field of view at selected wavelengths; (**C**) the SNR averaged over all wavelengths for selected regions of interest (ROI) that contained high fluorescence emission from one of the spectral components (labeled respectively in the legend); (**D**) the SNR averaged over an entire field of view for linearly unmixed images; (**E**) the SNR for the same regions of interest as C for linearly unmixed images. Open markers represent SNR in ROIs and closed markers represent SNR in entire field of view (FOV). The pinhole diameter is in airy disk (AD) units.

A range of confocal pinhole diameters was investigated (0.5 to 13.7 Airy disk (AD) units). Five sequential images were collected at each confocal pinhole setting, and the SNR was calculated as described in the methods. To obtain a composite SNR value, the SNR was averaged over all wavelengths. As expected, the average SNR increased with increasing pinhole diameter until maximal SNR was reached at ∼9 AD units (Figure 5).

The highest wavelength-averaged SNR for raw spectral images was 9. We observed the SNR was highly wavelength dependent (Figure 5B). To investigate the dependence of SNR on a specific fluorescence component (GFP, Hoechst, and autofluorescence) in the image, an ROI was selected for the region corresponding to a relatively pure composition of each spectral component. The SNR for selected ROIs also increased with increasing confocal pinhole diameter. The wavelength-averaged SNR for GFP and Hoechst regions was roughly twice the SNR for the whole image, as these regions contained higher fluorescence emission (Figure 5C). The SNR of linearly unmixed images was also calculated for the entire image ((Figure 5D) and for selected ROIs (Figure 5E)). The SNR for GFP calculated for the entire image was low compared to the Hoechst and autofluorescence. This is because GFP only occurred in small, localized regions (single cells), and hence, the pixel-averaged GFP signal was relatively weak. Because GFP was only present in selected regions of the image, an ROI was used to compare one of the selected regions of GFP to similar regions with high fluorescence emission from autofluorescence and Hoechst (see example in Figure 4). When the SNR was measured in selected ROIs (corresponding to high fluorescence emission from one component), unmixed GFP produced the highest SNR (∼60) while the autofluorescence produced the lowest SNR (∼12) (Figure 5E).

**Figure 6.**
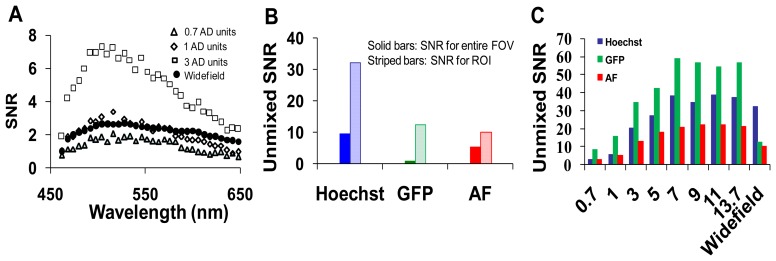
Signal-to-noise characteristics for the widefield system. (**A**) the signal to noise ratio (SNR) for the widefield was similar to the confocal microscope, at confocal pinhole diameters of 0.7 and 1 airy disk units; (**B**) the SNR for unmixed images and selected regions of interest (ROIs) within unmixed images; (**C**) a comparison of the confocal and widefield SNR for green fluorescent protein (GFP), Hoechst, and autofluorescence unmixed images (averaged over the full field of view).

We next collected image sets from the widefield microscope system and compared SNRs of the two microscope systems (Figure 6). The SNR for the hyperspectral widefield microscope was lower than that of the hyperspectral confocal microscope (Figure 6A). This is likely due to both the difference in detectors (EMCCD *vs.* PMT) and the higher background signal present in spectral images acquired on the widefield system. We observed that the SNR of Hoechst signal unmixed from widefield image stacks was higher than the SNR for the unmixed GFP or AF signals (Figure 6B). This is possibly because, due to the availability of excitation filters on the widefield system, Hoechst was excited with 360 nm, instead of the 405 nm excitation used with the confocal system. The unmixed SNRs for GFP, Hoechst, and autofluorescence images acquired using confocal and widefield systems were compared in Figure 6C.

#### 3.3.2. Laser Power and Photomultiplier Tube (PMT) Gain

**Figure 7.**
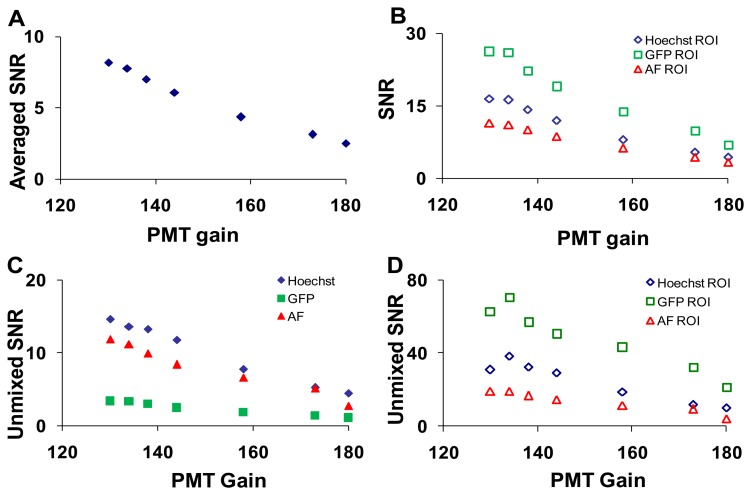
Increased photomultiplier tube (PMT) gain (at constant signal level, achieved by decreasing the laser intensity) resulted in decreased signal-to-noise ratio (SNR) for hyperspectral confocal images. (**A**) The wavelength-averaged SNR for an entire field of view; (**B**) the wavelength-averaged SNR for selected regions of interest (ROI) that contained high fluorescence emission from one of the spectral components (Hoechst, green fluorescent protein (GFP), autofluorescence (AF)); (**C**) the SNR averaged over an entire field of view for linearly unmixed images; (**D**) the SNR for the same ROI as B, but for linearly unmixed images.

To measure the effects of PMT gain on imager response, five sequential images were acquired at each setting (Table 1).The PMT gain and laser intensities were adjusted simultaneously to maintain a constant signal level (measured at 505 nm). As was performed for the confocal pinhole diameter analysis, the SNR was calculated at each PMT gain setting for the entire field of view, selected ROIs, and linearly unmixed images (Figure 7).

The effect of laser power on SNR was shown in Supplementary Figure 1. As expected, the wavelength-averaged SNR decreased with increasing PMT gain due to increased noise amplification (Figure 7A). When ROIs with high levels of a single fluorescent signature were analyzed the SNR for that fluorophore increased (Figure 7B). The SNR for the linearly unmixed GFP image (Figure 7C, solid green squares) was much lower than the SNR before unmixing (Figure 7B, open green square markers), due to averaging many non-GFP pixels (low or zero abundance) with only small regions (single cells) containing GFP pixels (high abundance). When ROIs were selected to isolate relatively pure areas of each component in the unmixed images, the SNR for all components increased greatly (Figure 7D).

#### 3.3.3. Arc Lamp Intensity and Electron-Multiplied Charge Coupled Device (EMCCD) Camera Gain

**Figure 8.**
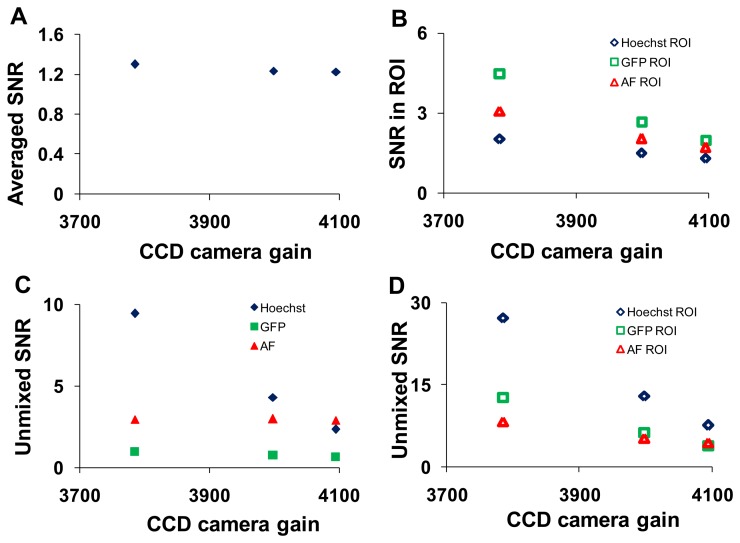
Electron-multiplied charge coupled device (EMCCD) gain (at constant signal level, achieved by decreasing the arc lamp intensity) resulted in decreased signal-to-noise ratio (SNR) for hyperspectral widefield images. (**A**) The wavelength-averaged SNR for an entire field of view; (**B**) the wavelength-averaged SNR for selected regions of interest (ROI) that contained high fluorescence emission from one of the spectral components (Hoechst, green fluorescent protein (GFP), autofluorescence (AF)); (**C**) the SNR averaged over an entire field of view for linearly unmixed images; (**D**) the SNR for the same ROI as B, but for linearly unmixed images.

Analogous study to altering the PMT gain on the hyperspectral confocal microscope was performed on the hyperspectral widefield microscope by adjusting the arc lamp intensity and the EMCCD camera gain. The wavelength-averaged SNR decreased with increasing charge-coupled device (EMCCD) gain (Figure 8A).

This trend remained for selected ROIs (corresponding to regions of relatively pure single components) in which the magnitude of the SNR was 2- to 3- fold higher (Figure 8B). The SNR increased following linear unmixing if the fluorescent component occupied a majority of the field of view (Figure 8C). When ROIs containing relatively pure compositions of single components were applied, the magnitude of the SNR increased for all components (Figure 8D). SNR data were plotted as a function of arc lamp intensity in Supplementary Figure 2.

Based upon these data, a confocal pinhole diameter of 4.9 AD units was selected for further studies. This diameter provides a compromise between signal-to-noise characteristics and the optical sectioning of the confocal image. In particular, because GFP-expressing endothelial cells are relatively thin, increasing the confocal pinhole diameter above 8 airy disk units resulted in no improvement in SNR for unmixed GFP (Figure 5E). To maximize SNR, a high laser intensity and low PMT gain were selected to perform photobleaching studies on the hyperspectral confocal microscope. Similarly, a maximal arc lamp intensity and EMCCD gain of 3500 were selected to perform photobleaching studies on the hyperspectral widefield microscope.

#### 3.3.4. Unmixing Error

**Figure 9.**
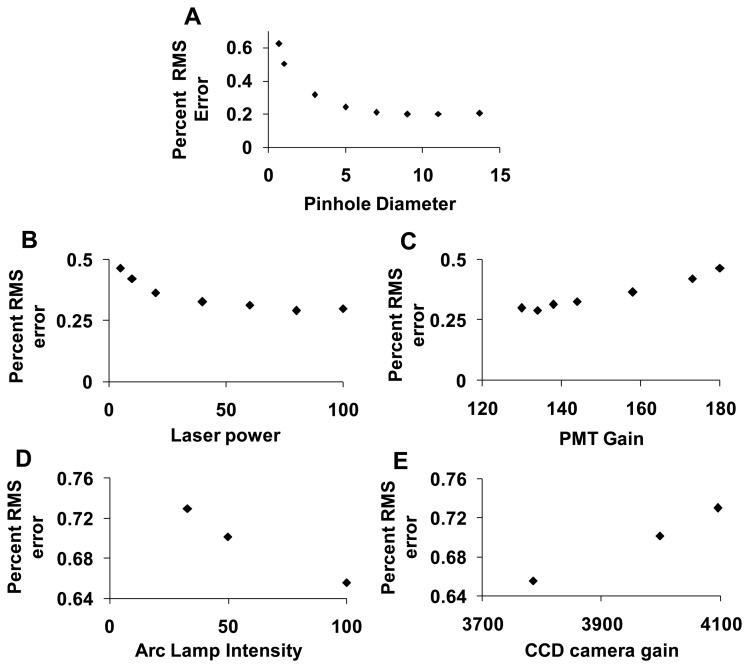
Effect of confocal pinhole diameter (**A**); confocal laser power (**B**); confocal photomultiplier tube (PMT) gain (**C**); widefield arc lamp intensity (**D**); and widefield electron-multiplied charge coupled device (EMCCD) camera gain (**E**) on the percent root-mean-square (RMS) error from linear unmixing.

To assess the error associated with spectral analysis, the root-mean-square (RMS) error from linear unmixing was calculated. The total RMS error was normalized by dividing by the RMS signal, as described in the Section 2.5.5, yielding the percent RMS error. The percent RMS error was calculated for each of the parameters described in the preceding sections. The percent RMS error decreased with increasing confocal pinhole diameter (Figure 9A), with increasing PMT gain (Figure 9C), and with increasing EMCCD gain (Figure 9E). The percent RMS error increased with increasing laser power (Figure 9B) and increasing arc lamp intensity (Figure 9D). Based on these results, a confocal pinhole diameter of 4 to 5 AD units seems appropriate to achieve a near-minimal percent RMS error of unmixing. Percent RMS error increased with PMT or EMCCD gain and decreased with laser power and arc lamp intensity, as would be expected. This highlights the need for sufficient illumination intensity when performing high dynamic-range hyperspectral imaging microscopy.

### 3.4. Photobleaching Studies

**Figure 10.**
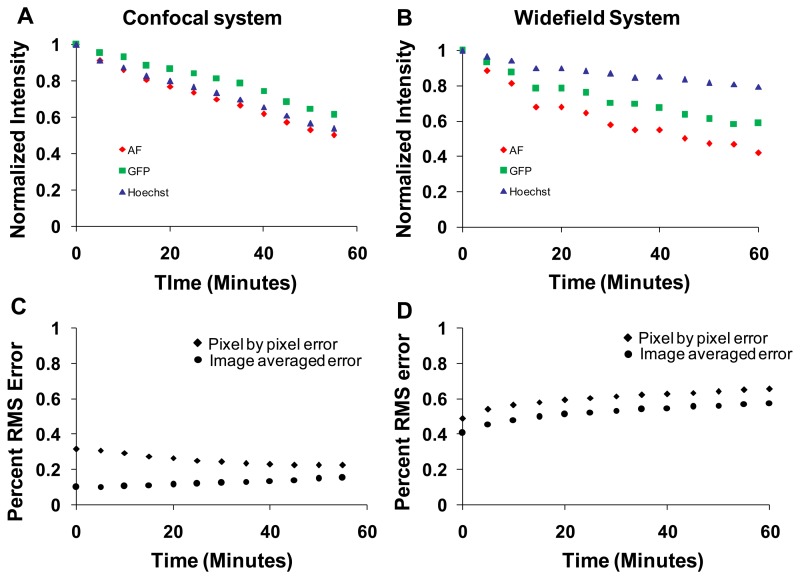
Photobleaching curves for average signal in each of the three spectral unmixed abundance images: autofluorescence (AF), green fluorescent protein (GFP), and Hoechst. Samples were illuminated continuously for 1 h on the hyperspectral confocal system (**A**) and hyperspectral widefield system (**B**); photobleaching rates for green fluorescent protein (GFP) and Hoechst were higher on the confocal system than on the widefield system, while photobleaching rates for autofluorescence (AF) were comparable between both systems. Percent RMS error of linear unmixing at different time points during photobleaching on the confocal system (**C**); and widefield system (**D**) during photobleaching.

Photobleaching effects were studied on both the confocal and widefield hyperspectral microscope systems. The sample was continuously illuminated for one hour and an image was acquired for every 5 min. Images were linearly unmixed, and the abundance (unmixed intensity) of each unmixed image was averaged over the entire field of view. Intensities for each component were normalized to the initial intensity and plotted as a function of time (Figure 10). The intensity of all fluorophores decreased due to photobleaching. The rate of photobleaching of all spectral components was relatively similar on the confocal system (Figure 10A). However, the rate of photobleaching of GFP and Hoechst was less than that of autofluorescence on the widefield system (Figure 10B). In addition, the rate of photobleaching measured on the widefield system was different from that measured on the confocal system. These differences are likely due to different excitation wavelengths used in each system and relative differences in excitation power (optical flux). The percent RMS error was also calculated at each time point. The image-averaged percent RMS error increased with bleaching time for both systems (measured by dividing the image-averaged RMS error by the image-averaged RMS signal, (Figure 10C,D, circles). The pixel-by-pixel percent RMS error decreased for the confocal system (Figure 10C, diamonds), but increased for the widefield system (Figure 10D, diamonds).

### 3.5. Theoretical Sensitivity Studies

**Figure 11.**
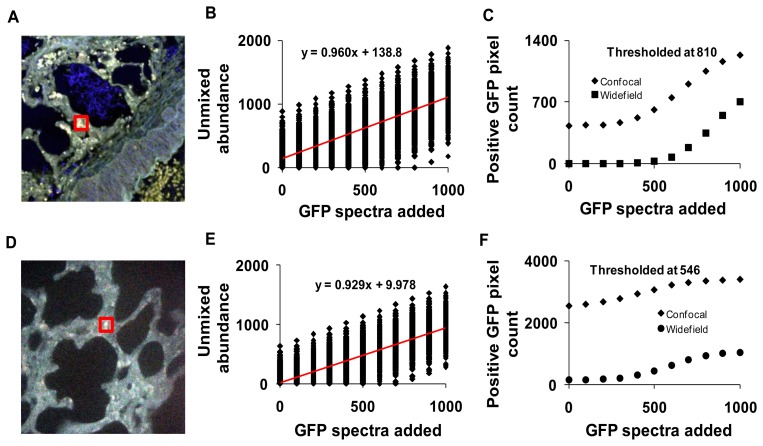
Known amounts of green fluorescent protein (GFP) spectra were added to a 30 × 30 pixel square region in the control (with no GFP and Hoechst) hyperspectral images to measure the theoretical sensitivity of the hyperspectral confocal and widefield microscope systems. Spectral images were then linearly unmixed and the GFP abundance for each pixel in the 30 × 30 region was plotted as a function of the GFP signal added (panels B and E, for confocal and widefield, respectively). The red line in panel B and E indicates the linear fit between the GFP added and GFP detected (e.g., at 1,000 pixels added almost all 1,000 pixels were detected). The minimum detection threshold for GFP-positive pixels was defined as the 98.5% quantile at 0% GFP added. Using this threshold (810 for confocal and 546 for widefield), the number of GFP-positive pixels in the entire image was measured and plotted as a function of the GFP signal added (panels C and F, for confocal and widefield, respectively).

The sensitivity of both the widefield and confocal hyperspectral imaging systems was estimated using a theoretical sensitivity study. A 30 × 30 pixel square region was selected in an autofluorescence control image from both the hyperspectral confocal and widefield imaging systems (Figure 11A,D). The spectrum of GFP was then added to the region, at varying peak intensity values from 0 to 1,000, in increments of 100 (A.U.). The dependence of unmixed GFP abundance on the signal intensity of GFP added was displayed using a theoretical sensitivity plot (Figure 11B, for confocal and Figure 11E for widefield system). A minimum detection threshold was defined as the 99.85% quantile for images with 0% GFP added (e.g., a pixel would have to have an unmixed GFP abundance of at least one standard deviation above the mean abundance for the image with 0 GFP signal added, in order to be detected). All pixels in the image were then classified as positive or negative using this threshold value. For comparison, images from both systems were analyzed using the threshold from both the confocal (810 unmixed abundance, Figure 11C) and the widefield (546 unmixed abundance, Figure 11F) systems.

### 3.6. Optimization of Hyperspectral Imaging Systems

The hyperspectral assay in this study was designed to identify the GFP-expressing pulmonary microvascular endothelial cells in highly autofluorescent lung tissue sections [9]. Table 2 summarizes the results of the parametric sensitivity study and the optimal parameters required for this assay.

Optimal equipment settings and acquisition parameters will depend upon both the type of hyperspectral imaging system and the experimental design. Important factors in determining optimal acquisition parameters include: the spectral excitation and emission characteristics of the fluorophores and any autofluorescence, the quantum efficiency of each fluorophore, the variation in concentrations of fluorophores, and the temporal resolution required. General optimization settings that are applicable to many types of fluorescence hyperspectral imaging experiments are tabulated in Table 3.

## 4. Discussion

We have developed an approach to compare and characterize hyperspectral imaging systems. In this study, we used a widefield system that utilizes an acousto-optic tunable filter (AOTF) and camera and a confocal system grating and 32-channel photomultiplier tube (PMT). We performed background subtraction and flat spectral correction on images acquired from both systems. We then performed parametric sensitivity studies to characterize both systems and to determine optimal values for operating parameters. In addition, we measured the rate of photobleaching for the fluorescent components in the sample. Finally, we performed theoretical sensitivity studies to measure the sensitivity and specificity of both systems for detecting GFP in the presence of lung autofluorescence. Results from this study indicate that hyperspectral microscopy, with linear unmixing, can reliably detect low fluorophore signal intensities, even in the presence of high autofluorescence. However, caution should be used when performing multifluor measurements, as differential photobleaching can occur.

In this study, all images were corrected to remove nonspecific background and to achieve a flat spectral response. Nonspecific background can be attributable to several factors, including stray light, out-of-band cross-talk, and excitation-emission cross-talk. In hyperspectral imaging, nonspecific background may be higher than traditional single-channel imaging, due to the limited out-of-band rejection provided by tunable filters [17] or dispersive elements, in comparison to standard interference filters. It should be noted that spectral background subtraction, as implemented here, helps to correct for nonspecific background that is uniform across the sample. Alternative approaches could be developed to correct for spatially-dependent background, if that background signal were reproducible.

After background subtraction, all images were corrected to a flat spectral response. For the hyperspectral widefield system, spectral correction was performed using a US National Institute of Standards & Technology (NIST)-traceable lamp to correct for the wavelength-dependent response of the tunable filter and EMCCD detector. Flat spectral correction revealed that wavelengths above 550 nm were transmitted more efficiently than lower wavelengths. The hyperspectral confocal system is equipped with a NIST-traceable detector and no further spectral correction was performed. After correction, both systems produced consistent spectra from single fluorophore-labeled samples (compare GFP spectra in panels A and B of Figure 3).

Spectrally-corrected image sets were used to construct spectral libraries that contained the end-members (GFP, autofluorescence and Hoechst) for unmixing. Separate libraries were constructed for the hyperspectral widefield and confocal microscope systems. While the Hoechst and GFP emission spectra were similar and matched those predicted by literature [24–26], we observed that the emission peak for autofluorescence was different for widefield (505 nm, Figure 3A) and confocal (545 nm, Figure 3B) systems. These spectral differences may be attributed to the use of different excitation wavelengths: a 430/24 nm excitation filter on the widefield system and a 457 nm laser on the confocal microscope. Because lung autofluorescence is due to the presence of multiple endogenous fluorophores (e.g., collagen, elastin), and because each fluorophore has a characteristic excitation spectrum [27], the relative excitation of each species of fluorophore within a given pixel will vary with excitation wavelength. Hence, the peak wavelength of the bulk autofluorescence emission spectrum depends on the excitation wavelength. In addition, the laser excitation of the confocal system provides single wavelength excitation at two laser lines (405 and 458 nm). These laser lines were selected to allow excitation of all fluorescent species in the sample. The widefield system achieves a similar effect by providing a broad wavelength-band excitation. However, to make these excitation profiles ideally comparable, an identical laser excitation profile would need to be implemented on the widefield system. Although beyond the scope of this study, this could be achieved by splitting the excitation beam path from the confocal system and using the same laser excitation for the widefield system. If identical excitation profiles were available, the results for SNR and RMS error would likely be somewhat altered for the widefield system (6 and 7). Hence, these results demonstrate performance capabilities for comparable—but not identical—operating conditions on each system. Because of excitation-dependent effects, and because equipment settings and system configurations can vary, care should be taken when defining standard databases of spectra that will be used for unmixing because the emission spectra for complex samples (such as tissue) may depend upon the excitation wavelength(s) and the acquisition parameters (e.g., spectral step size, filter bandwidth).

A parametric study was performed to characterize both hyperspectral imaging systems. The operating parameters that were varied on the confocal system include the confocal pinhole diameter, PMT gain, and laser power. The parameters varied on the widefield system include the EMCCD gain and arc lamp intensity. The signal-to-noise ratio (SNR) and root-mean-square error (RMS error) from linear unmixing were measured at multiple settings for each parameter to determine the noise characteristics of each system and the error associated with the linear spectral unmixing. In general, the confocal hyperspectral imaging system offered higher SNR than the widefield system. This could be due the differences in the detectors (EMCCD in widefield and PMT in confocal) and due to the subtraction of a high nonspecific background signal from widefield images. In all cases, linear unmixing resulted in increased SNR. This is true for most hyperspectral imaging systems, as the narrow bandwidth of spectral filtering results in poor photon statistics at each spectral band (especially for fluorescence images), but linear unmixing (and other spectral analysis algorithms) take data in many bands into account when calculating unmixed endmember images, thereby increasing the signal quality of each unmixed endmember image. In addition, the SNR is varied between wavelengths in raw spectral image data and between endmembers in linearly unmixed images. Both of these variations are attributed to real variations in fluorophore concentration within the sample, as wavelength bands with high SNR corresponded to peak emission wavelengths of the endmembers in the library.

The accuracy of spectral analysis algorithms is directly related to the noise characteristics in the raw spectral image. This was demonstrated by the markedly lower percent RMS error associated with linear unmixing of images from the hyperspectral confocal system, as compared to the hyperspectral widefield system. The accuracy of spectral analysis also depends on the signal strength (fluorescence intensity) and variability, or coefficient of variance (CV), of the signal. To demonstrate this, we calculated mean fluorescence intensities and CVs for GFP, Hoechst, and AF for the ROIs in the image and found that the CV for GFP, Hoechst and AF was 0.008, 0.014, and 0.02 respectively. While all of these values represent relatively low CVs, it is interesting to note that there is roughly a five-fold difference in CVs between the different fluorophores in the sample. This indicates that some fluorophore concentrations are much more consistent than others, which is likely to be expected in most imaging assays where different probes or autofluorescent molecules are distributed heterogeneously. The SNR for raw spectral images from the confocal system ranged from 5 to 30 and the corresponding percent RMS unmixing error ranged from 20% to 60% while the SNR for raw spectral images from the widefield system ranged from 1.5 to 5 and the corresponding % RMS unmixing error ranged from 65% to 75%. Based on the SNR and percent RMS error data, we selected a pinhole size of 4.9 AD units to be an appropriate compromise between signal strength and photobleaching (discussed below). As expected, the percent RMS error had an inverse relationship with the illumination intensity and was directly related to detector gain. These results suggest that a high illumination intensity and low detector gain offer improved performance for high dynamic range hyperspectral microscopy, when acquisition times and/or photobleaching are not constrained.

Photobleaching studies were performed by illuminating the sample for an hour on both imaging systems while acquiring spectral image sets intermittently. Photobleaching resulted in lower image quality due to the lower emitted photon flux. The rates of photobleaching differed between imaging systems, and also differed between spectral components (Figure 10). The rate of photobleaching of autofluorescence was comparable between systems, however, the rate of photobleaching of Hoechst and GFP was higher on the confocal system compared to the widefield system. This may be due to the intense illumination power (laser intensity) used in the confocal microscope. The difference between the use of monochromatic excitation sources (laser sources) and an arc lamp with band-pass excitation filter may also contribute to differences in photobleaching between systems. To better understand photobleaching differences between both systems, we measured the light intensity at the objective for each system using an integrating sphere and fiber-coupled, NIST-calibrated spectrometer. The illumination intensity for the widefield system (386 μW for 430 nm excitation and 70.5 μW for 360 nm excitation) was high compared to that of the confocal (5.86 μW for the 458nm laser and 1.8 μW for 405 nm laser line). However, the illumination in the widefield system is dispersed over the entire field of view for the duration of the spectral image scan, whereas in the confocal system, the full illumination is provided to each pixel, albeit for a very short dwell time. Hence, differences in photobleaching may be due not just to total optical irradiance over time (dose) but also to the magnitude of irradiance and the localized fluorophore concentration in each region being irradiated.

To compare static sensitivity response, theoretical sensitivity studies were performed on both hyperspectral microscope systems. Results from these studies allowed us to measure the sensitivity and specificity of each system for detecting GFP in the presence of lung autofluorescence. The theoretical sensitivity studies were performed by adding known amounts of GFP spectra to a hyperspectral image of lung autofluorescence. The resultant image was then linearly unmixed and the abundance of GFP within pixels to which GFP was added was quantified. GFP abundance data were plotted as a function of the level of GFP added and fit with a linear trend. The slopes of the sensitivity plots for both systems were very similar, indicating that both systems responded ideally to increases in GFP signal level (Figure 11B,E). A minimum detection threshold was then defined as the 99.85% quantile (of abundance) when no GFP signal was added to the image. This detection threshold was used to determine the number of GFP-positive pixels in unmixed images. Results indicate that the widefield hyperspectral system produced fewer false positive pixels (0.06%, or 157 pixels in 262,144 pixels) than the confocal hyperspectral system (0.96%), when thresholded at the 99.85% quantile from the widefield system (546 GFP abundance units, Figure 11C). The number of false positives detected decreased with increasing threshold value for both the widefield (0.0004% false positives) and confocal (0.16% false positives) systems (Figure 11D). These results indicate that the widefield hyperspectral system was more specific (fewer false positives (type I errors)) than the hyperspectral confocal system for detecting GFP, even though the confocal system offered improved signal-to-noise characteristics. However, the confocal system detected all the GFP positive pixels with fewer false negatives (type II errors) than the widefield microscope.

The differences in sensitivity and specificity that can be measured using the theoretical sensitivity study are likely due to several factors, several of which have already been discussed. Key factors include the SNR, the mixing ratios (spatial heterogeneity) of fluorophores in the sample, the excitation characteristics, and the similarity between the GFP emission spectrum and the emission spectra of other fluorophores in the sample. As can be seen by comparing Figures 5 and 11, although the SNR of raw spectral images acquired on the confocal system was higher than those acquired on the widefield system (Figure 5), the variance of unmixed GFP abundances is higher for images acquired on the confocal system (shown as the spread of unmixed GFP abundances in Figure 11B,E). This could be due to the alternative excitation profile (laser lines) of the confocal system or the shift in the autofluorescence spectrum that is associated with a different excitation (Figure 3). Hence, several factors are involved in determining the actual ability to detect GFP-positive pixels and discriminate among GFP-positive and GFP-negative pixels in the image. Because of the multiple factors involved in determining sensitivity and specificity, it is likely that these differences would be present even in hyperspectral microscopy systems that use similar spectral sampling techniques—such as AOTFs and liquid-crystal tunable filters (LCTFs), both of which use a sequential spectral sampling method, allow roughly 20%–35% transmission efficiency, can be adjusted to have similar spectral bandwidths, and both of which can be implemented using a CCD or EMCCD camera. While we do not currently have two such systems available in our laboratory, configuring two such identical systems for comparison (with the exception of spectral filtering technology) could be a beneficial future study for elucidating the effects of spectral filtering on accurate signal detection. In addition, it may also be possible to modify this theoretical sensitivity study approach to allow rapid evaluation and comparison of equipment settings (such as varying laser line wavelength, detector gain, spectral sampling interval, or integration time) and their effect on the final unmixing sensitivity or classification specificity.

There are several alternative technologies for acquiring hyperspectral microscopy data that utilize either diffractive optics such as a prism [28,29] or grating [30,31], Fourier transform theory [32], or alternative tunable filter technologies such as liquid-crystal tunable filters [33] and thin-film tunable filters [10,11]. Most widefield prism- and grating-based systems utilize a push-broom scanning approach wherein a very high number of bands (>100) are acquired at the expense of not sampling spatial data in the XY plane simultaneously. We expect that signal to noise characteristics for these systems would be similar to the widefield system tested in this work. However, photobleaching kinetics may vary depending on the scanning method and the percent of fluorescence emission that is detected simultaneously (e.g., push broom methods typically detect all spectral bands simultaneously whereas sequential methods discard fluorescence emission outside of the band being measured). For any of these technologies, the approach outlined in this paper could be applied to investigate the relationship between instrument settings, signal-to-noise characteristic of the image, error associated with linear unmixing, photobleaching kinetics, and theoretical sensitivity of the system and resulting fluorescence measurements.

## 5. Conclusions

Hyperspectral imaging and analysis approaches depend highly on the signal and noise characteristics of the hyperspectral imaging system. In this study, we compared the parametric response of two hyperspectral imaging systems—one based on a widefield fluorescence microscope and one based on a confocal fluorescence microscope. We found that it was necessary to carefully select system parameters such as the confocal pinhole, detector gain, illumination intensity, and exposure time in order to optimize the performance of the system. In addition, when using time-lapse or live-cell imaging, it may be necessary to achieve a compromise between imaging performance and the extent of error that can be tolerated from differential photobleaching and loss of signal strength. Both hyperspectral microscopy systems were found to be linearly sensitive to additions of a single signal, although the specificity varied widely between systems. Thus, optimization of system parameters is likely more important for hyperspectral imaging microscopes than for traditional, single-band fluorescence microscopes. The methods described in this study serve as an approach for characterizing and comparing alternative hyperspectral imaging equipment configurations or system parameters, and can be applied to optimize hyperspectral imaging system performance for different fluorescence imaging assays.

## Figures and Tables

**Table 1.** At each setting of 457.9 nm excitation, the other laser intensity was changed, such that the ratio between the two laser intensities remains the same. Photomultiplier tube (PMT) gain was adjusted accordingly to utilize the complete dynamic range.

**Excitation Laser Intensities (%)**		**PMT Gain**

**457.9 nm Laser**	**405 nm Laser**	
5	0.2	180
10	0.3	173
20	0.7	158
40	1.3	144
60	2	138
80	2.7	134
100	3.3	130

**Table 2.** Optimal parameters that were determined for detecting green fluorescent protein (GFP) emission in mixed samples with high autofluorescence.

**Experiment/Parameter**	**Widefield Microscope**	**Confocal Microscope**
Confocal pinhole	NA	4.9 Airy disk units
Illumination power	Arc lamp–100%	405 laser–60% 57 laser–2%
Detector gain	EMCCD–3900	PMT–138
Photobleaching	Similar bleaching rates in widefield and confocal for AF. Hoechst and GFP-Less	Hoechst and GFP comparatively high
SNR	Low SNR due to high background signal (SNR:2–3)	Medium SNR (SNR: 3–10)
RMS error	High	Low
Sensitivity	Linear: 0.929 slope (GFP detected/GFP added)	Linear: 0.96 slope (GFP detected/ GFP added)
Specificity	0.06% Type I errors	0.16 % Type I errors

**Table 3.** General classes of fluorescence hyperspectral imaging assays and optimal operating parameters.

**Type of Spectral Assay**	**Optimization Settings**
**Case I**: High signal, high speed imaging	[Fluorophore A]∼ [Fluorophore B] High laser power Short acquisition time Low averaging Results in high SNR
**Case II**: High signal, variable concentration	[Fluorophore A] ≫ [Fluorophore B] Maximize dynamic range High laser power Short acquisition time Results in high SNR
**Case III**: Low signal (sensitive), high speed imaging	Low laser power Short acquisition time Low SNR Balance: - temporal resolution - photobleaching - SNR effects unmixing accuracy (RMS error)
**Case IV**: Low signal, variable concentration	Maximize dynamic range Low laser power Long acquisition time Results in low SNR Balance with photobleaching kinetics

## Supplementary Files

Supplementary File 1 Supplementary Information (PDF, 24 KB)
