# Supplementary material for: An Approach for Characterizing and Comparing Hyperspectral Microscopy Systems
Source: Sensors (Basel). 2013 Jul 19;13(7):9267–93. doi: 10.3390/s130709267 (PMC3758648; doi:10.3390/s130709267)
Supplement: Supplementary File 1 — Supplementary Information (PDF, 24 KB) [file sensors-13-09267-s001.pdf]

OPEN ACCESS

sensors

ISSN 1424-8220

www.mdpi.com/journal/sensors

Supplementary Information

An Approach for Characterizing and Comparing Hyperspectral Microscopy Systems. *Sensors* 2013, *13*, 9267-9293

Naga S. Annamdevula [1], Brenner Sweat [1], Peter Favreau [1], Ashley S. Lindsey [2], Diego F. Alvarez [2,3,4], Thomas C. Rich [2,3,5] and Silas J. Leavesley [1,2,3,*]

[1] Department of Chemical and Biomolecular Engineering, University of South Alabama, 150 Jaguar Dr., SH 4129, Mobile, AL 36688, USA; E-Mails: nsa801@jagmail.southalabama.edu (N.S.A.); ws1001@jagmail.southalabama.edu (B.S.); pff601@jagmail.southalabama.edu (P.F.)

[2] Department of Pharmacology, University of South Alabama, 5851 USA Dr. N., Mobile, AL 36688, USA; E-Mails: aes901@jagmail.southalabama.edu (A.S.L.); dalvarez@southalabama.edu (D.F.A.); trich@southalabama.edu (T.C.R.)

[3] Center for Lung Biology, University of South Alabama, 5851 USA Dr. N., Mobile, AL 36688, USA

[4] Department of Internal Medicine, University of South Alabama, 5851 USA Dr. N., Mobile, AL 36688, USA

[5] College of Engineering, University of South Alabama, 150 Jaguar Dr., Mobile, AL 36688, USA

***** Author to whom correspondence should be addressed; E-Mail: leavesley@southalabama.edu; Tel.: +1-251-460-6160; Fax: +1-251-460-1485.

Figure S1. The effect of laser power on signal to noise ratio (SNR). The averaged SNR ratio increased with the laser power (**A**). The SNR for ROIs of each component (GFP, Hoechst, and AF) in the image prior to unmixing (**B**). The SNR for the whole unmixed images (**C**). The SNR for ROIs of each component in the unmixed images (**D**).

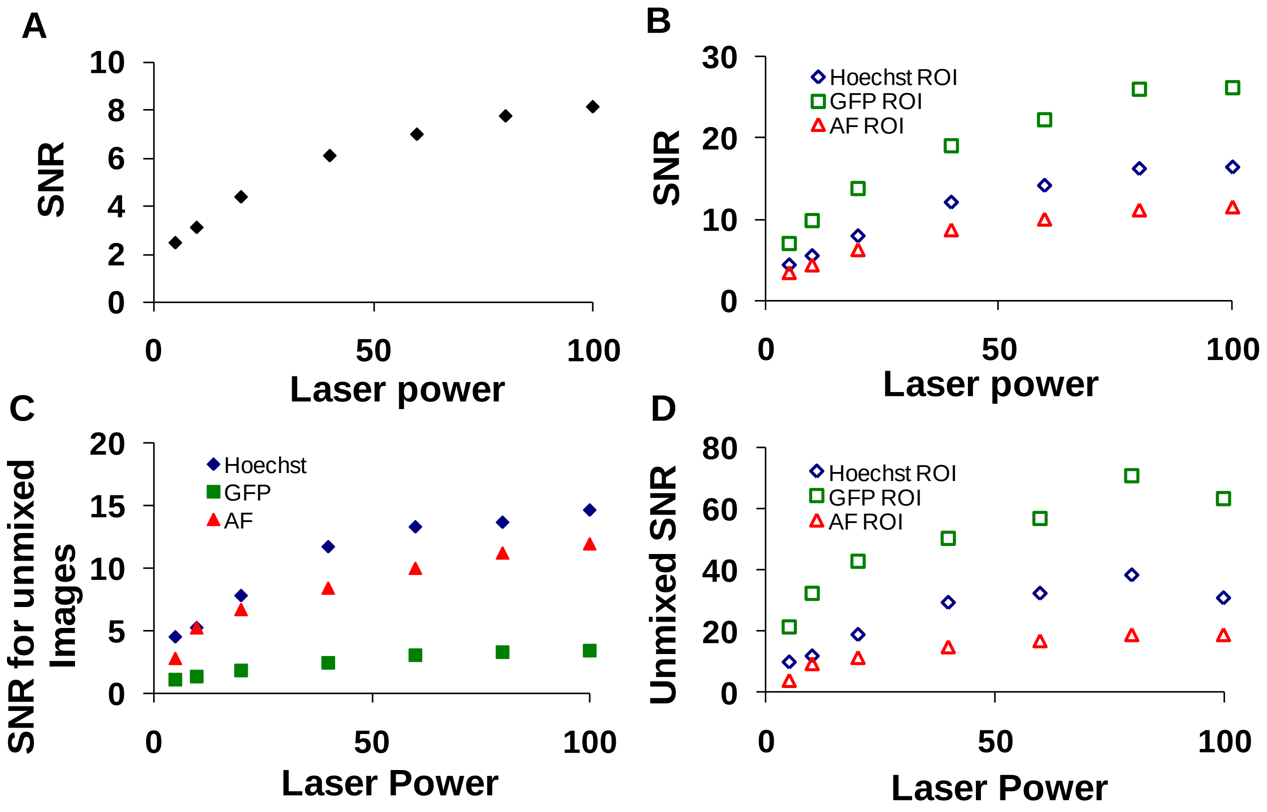

Figure S2. The effect of arc lamp intensity on signal to noise ratio (SNR). The SNR ratio averaged over all the wavelengths increased with the arc lamp power (**A**). The SNR for ROIs of each component (GFP, Hoechst, and AF) in the image prior to unmixing (**B**). The SNR for the whole unmixed images (**C**). The SNR for ROIs of each component in the unmixed images (**D**).

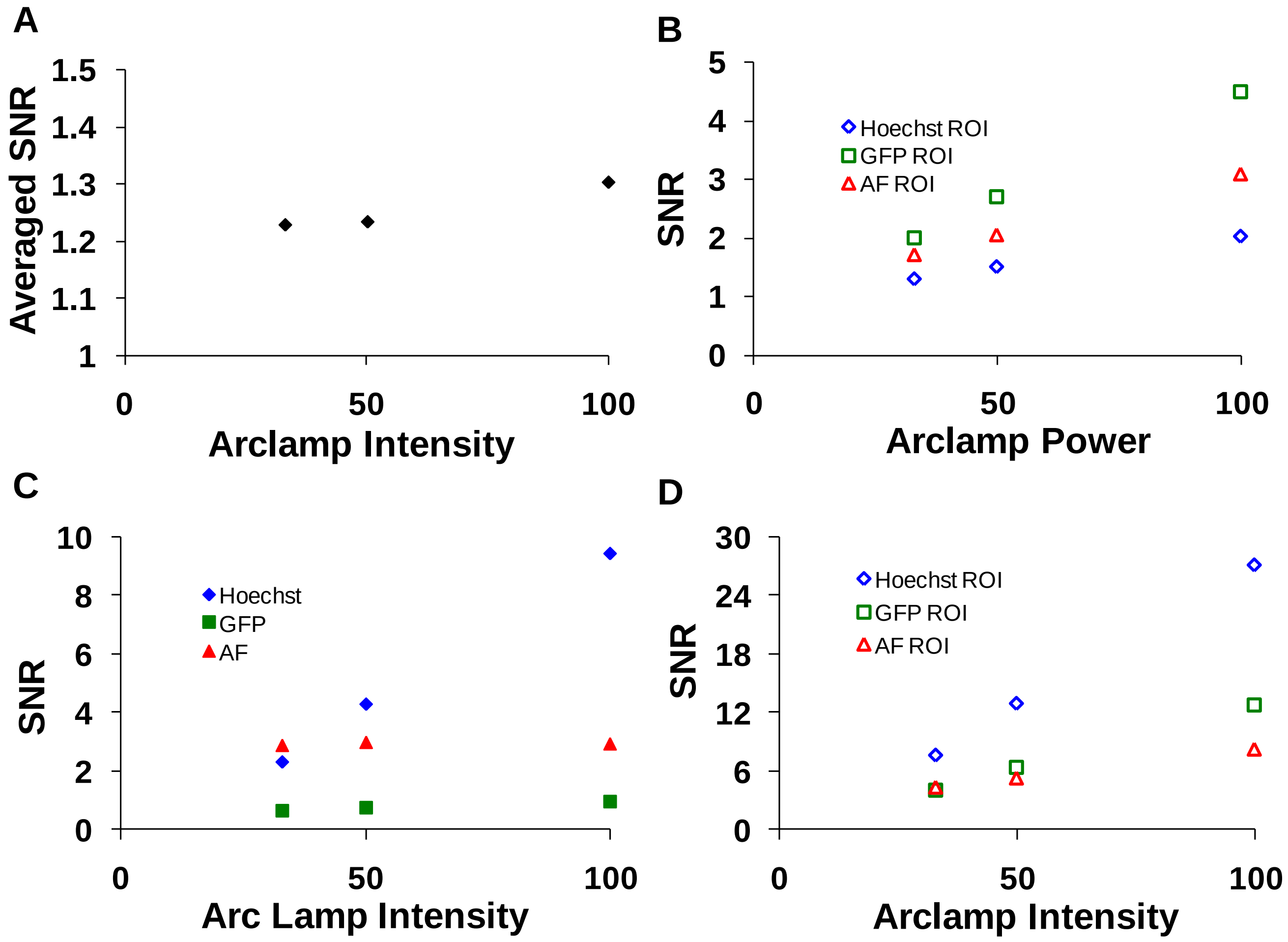

© 2013 by the authors; licensee MDPI, Basel, Switzerland. This article is an open access article distributed under the terms and conditions of the Creative Commons Attribution license (http://creativecommons.org/licenses/by/3.0/).
